# The Emerging Role of Innate Lymphoid Cells (ILCs) and Alarmins in Celiac Disease: An Update on Pathophysiological Insights, Potential Use as Disease Biomarkers, and Therapeutic Implications

**DOI:** 10.3390/cells12141910

**Published:** 2023-07-21

**Authors:** Angela Rizzi, Mario Di Gioacchino, Luca Gammeri, Riccardo Inchingolo, Raffaella Chini, Francesca Santilli, Eleonora Nucera, Sebastiano Gangemi

**Affiliations:** 1 UOSD Allergologia e Immunologia Clinica, Dipartimento di Scienze Mediche e Chirurgiche Addominali ed Endocrino Metaboliche, Fondazione Policlinico Universitario A. Gemelli IRCCS, 00168 Rome, Italy; angela.rizzi@policlinicogemelli.it (A.R.); raffaella.chini01@gmail.com (R.C.); eleonora.nucera@policlinicogemelli.it (E.N.); 2 Institute for Clinical Immunotherapy and Advanced Biological Treatments, 65100 Pescara, Italy; 3 Center for Advanced Studies and Technology, G. d’Annunzio University, 66100 Chieti, Italy; francesca.santilli@unich.it; 4 Department of Clinical and Experimental Medicine, School and Operative Unit of Allergy and Clinical Immunology, University of Messina, 98125 Messina, Italy; lucagammeri@outlook.com (L.G.); gangemis@unime.it (S.G.); 5 Pulmonary Medicine Unit, Department of Neurosciences, Sense Organs and Thorax, Fondazione Policlinico Universitario A. Gemelli IRCCS, 00168 Rome, Italy; riccardo.inchingolo@policlinicogemelli.it; 6 Medicina e Chirurgia Traslazionale, Università Cattolica del Sacro Cuore, 00168 Rome, Italy

**Keywords:** celiac disease, ILC, intestinal epithelial cells, alarmins, TSLP, IL-33, HMGB1, inflammation, therapeutic strategies

## Abstract

Celiac disease (CD) is an intestinal disease that develops in genetically predisposed individuals and is triggered by the ingestion of gluten. CD was considered a Th1-disease. Today, the role of Th17, IL-21, and IL-17A lymphocytes is well known. Inflammation is regulated by the activity of gluten-specific CD4+ T lymphocytes that produce pro-inflammatory cytokines, including IFN-γ, TNF-α, and IL-21, perpetuating the Th1 response. These cytokines determine an inflammatory state of the small intestine, with consequent epithelial infiltration of lymphocytes and an alteration of the architecture of the duodenal mucosa. B cells produce antibodies against tissue transglutaminase and against deamidated gliadin. Although the role of the adaptive immune response is currently known, the evidence about the role of innate immunity cells is still poorly understood. Epithelial damage determines the release of damage-associated molecular patterns (DAMPs), also known as alarmins. Together with the intestinal epithelial cells and the type 1 innate lymphoid cells (ILC1s), alarmins like TSLP, IL-33, and HMGB1 could have a fundamental role in the genesis and maintenance of inflammation. Our study aims to evaluate the evidence in the literature about the role of ILCs and alarmins in celiac disease, evaluating the possible future diagnostic and therapeutic implications.

## 1. Introduction

Celiac Disease (CD) is a chronic enteropathy triggered by eating gluten in genetically predisposed individuals and characterized by multiple pathways of mucosal damage in the small intestine [1]. The leukocyte histocompatibility antigen genes HLA-DQ2 and HLA DQ8 are the most implicated genes for predisposition to this disorder [2]. Genome-wide association studies (GWASs) also identified non-HLA genetic factors, most of which are related to immuno-biologically relevant genomic regions like the IL2–IL21 region and some genes controlling immune responses (*CCR3*, *IL12A*, *IL18RAP*, *RGS1*, *SH2B3*, and *TAGAP*) [3,4]. Environmental factors act on the genetic substrate; this event is necessary for the disease to manifest itself. Among these, the main one is dietary gluten, particularly gliadins (key components of gluten).

Until recently, CD was considered a Th1-predominant disease. Gluten-specific CD4+ T cells produce pro-inflammatory cytokines such as interferon (IFN)-γ, tumor necrosis factor (TNF)-α, and interleukin (IL)-21, perpetuating the Th1 response and stimulating the production of antibodies against tissue transglutaminase (tTG) and against deamidated gliadin [5]. 

This pro-inflammatory environment determines an inflammatory state of the small intestine, with consequent epithelial lymphocytic infiltration and an alteration of the architecture of the duodenal mucosa [6]. 

Recent studies demonstrated the role of innate lymphoid cells (ILCs) in the pathogenesis of intestinal inflammatory diseases [7]. ILCs are lymphoid lineage cells that do not express the antigen receptors usually expressed by T and B cells and belong to the cell class of innate immunity [7]. 

ILCs are diffused in the liver and the mucosa of the gastrointestinal tract [8]. Here, they play a key role in intestinal homeostasis and regulating immune and inflammatory responses, modulating cytokine secretion [9].

Epithelial damage will release damage-associated molecular patterns (DAMPs), proteins associated with sterile inflammation [10], which could initiate and perpetuate the inflammatory process. Alarmins are proteins or peptides belonging to a subset of endogenous DAMPs that interact with chemotactic and pattern recognition receptors (PRRs) (e.g., Toll-like receptors) to stimulate the immune response [11]. Alarmins are released following degranulation, damage, or cell death, stimulating immune cells in host defense [12]. 

The potential role of alarmins in the pathogenesis of diseases like autoimmune diseases and food allergies is well known [13,14]. For example, TSLP and IL-33 play a fundamental role in the development of food tolerance and their alteration could lead to the genesis of adverse food reactions [14]. Numerous studies have highlighted the role of alarmins in the genesis of other pathologies of the gastrointestinal system. Indeed, IL-33, mainly produced by intestinal epithelial cells and mononuclear cells of the lamina propria, stimulates the production of pro-inflammatory cytokines during active inflammation and promotes Th2 and Th17 adaptive immune responses [15]. 

**Figure 1:**
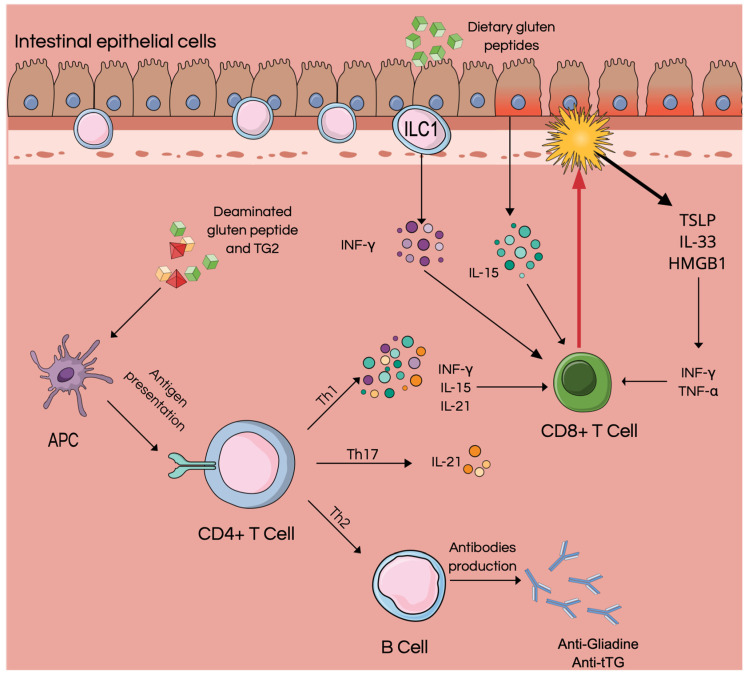
Pathogenetic mechanisms of celiac disease. In genetically predisposed subjects, gliadin-derived peptides determine the activation of CD4+ T cells; the development of a Th1 and Th17 response; and the consequent production of INF-γ, IL-15, and IL-21 [5]. The proinflammatory environment contributes to epithelial damage, resulting in the release of other proinflammatory cytokines and alarmins such as TSLP, IL-33, and HMGB1.

Our work aims to investigate the role of innate immunity and its key player in the genesis and maintenance of CD, focusing on the role of intestinal epithelial cells, ILCs, and alarmins (Figure 1). We, therefore, carried out a literature review to understand the potential diagnostic–therapeutic role of alarmins.

## 2. Intestinal Epithelial Cells

The inflammatory environment in celiac pathology favors the damage of the epithelial cells and all that follow, such as maintenance of the inflammatory process, alterations of the barrier, and villar atrophy.

The changes in enterocyte morphology are a direct consequence of IFN-γ-dependent inflammation; this determines a reduction in tubulin tyrosine ligase (TTL) and an increased expression of vasohibin 2 (VASH2) with a consequent increase in the amount of stable detyr-tubulin and alteration of the cell shape [16]. 

Purple et al., in their study of bioptic samples of duodenal mucosa and organoids (miniaturized models of organs), observed an increase in IL-1-β and IL-6 expressed by the enterocytes of patients with CD, both in the acute phases and in remission; moreover, CD organoids were shown to be more sensitive to inflammatory stimuli induced by Toll-like receptor ligand loxoribine (Lox) and gliadin peptide P31-43. These results demonstrate an underlying low-grade inflammation of the enterocytes of CD patients [17].

The inflammatory environment also determines an altered gene expression at the enterocyte level. Bracken et al. evaluated the gene expression alterations of the enterocytes of patients with CD, finding 102 altered gene expressions compared to healthy subjects. Some genes implicated in reducing apoptosis are overexpressed in these subjects, such as the glucocorticoid-induced TNFR-related gene (TNFRSF18) and erythropoietin. At the same time, the protein tyrosine kinase 2 beta (PTK2B), which is instead anti-apoptotic, is downregulated. Five genes involved in the immune response were also identified, including MAP/microtubule affinity regulator kinase 2 (MARK2). MARK2 plays a role in maintaining homeostasis of the immune system, and in celiac enterocytes, there is a reduced expression [18]. Braken and her team identified other altered genes responsible for cell proliferation, transport, and intestinal mucosal integrity.

IL-15 is produced and released by intestinal epithelial cells and is thought to be the link between intestinal epithelial cells and the intestinal immune system [19]. IL-15 is increased in the serum and intestinal mucosa of subjects with CD, both in active disease and remission phases [20]. In 2006, Di Sabatino and co-workers demonstrated the central role of IL-15 in the modulation of the innate immune response to gliadin in patients with CD. IL-15 overexpression in active disease stimulates inflammatory Th1 cells, promoting epithelial damage [21]. This cytokine is the most potent stimulator of ILCs; indeed, IL-15 promotes ILC proliferation and the release of IFN-γ [22]. The gliadin P31-43 peptide can initiate the innate immune response through the action mediated by IL-15 [20].

Gliadin and P31-43 peptide alter the epithelial growth factor receptor (EGFR) signaling pathway and thus the proliferation of enterocytes in CD patients [23]. Nanayakkara and co-workers found an increase in proliferative activity and the EGFR/ligand system in the enterocytes of these patients, associated with an increase in phosphorylated downstream signaling molecule Extracellular Signal-Regulated Kinase (ERK). Thus, P31-43 peptide promotes CD enterocyte proliferation in an EGF- and IL-15-dependent manner, enhancing the EGFR-ERK pathway and resulting in crypt hyperplasia and tissue remodeling of the CD intestine; this does not occur in healthy subjects [23].

## 3. ILC

Innate lymphoid cells (ILCs) are lymphocytes, mainly located at the tissue level, that lack the diverse antigen receptors typically expressed on T and B cells [7]. These cells mimic the function of T helper cells (Th)1, Th2, and Th17 and have therefore been classified as ILC1, ILC2, and ILC3, respectively [24]. Since 2018, ILCs have been divided into five categories: NK cells, ILC1, ILC2, ILC3, and lymphoid tissue inducer cells (LTi) [7].

ILCs appear to have multiple roles in our bodies. Indeed, it is now clear that all ILC subsets play a role in innate immune responses to viruses, bacteria, fungi, and parasites; conversely, they also play a role in maintaining intestinal homeostasis, limiting inappropriate immune responses to commensal microorganisms [25,26]. ILC2 and ILC3 contribute to tissue remodeling. Furthermore, the chronic activation of ILC may play a role in the pathogenesis and maintenance of chronic inflammatory diseases [7]. 

ILC1 is responsible for producing IFN-γ, ILC2 produces IL-5 and IL-13, and ILC3 produces IL-17A and IL-22 [7].

Recent studies in mice and humans have demonstrated the role of ILCs in the pathogenesis of chronic intestinal inflammation and the development of colitis-associated cancer in patients with inflammatory bowel disease (IBD) [26]. 

Therefore, ILCs could have a role in the pathogenesis of other intestinal pathologies, such as CD. However, there are few studies in this regard, and the mechanisms still need to be fully understood.

Marafini et al. evaluated the expression of ILCs and pro-inflammatory cytokines in the duodenal mucosa of patients with CD. They demonstrated no difference between CD patients and controls in the percentage of total ILC in the mucosa. Nevertheless, ILCs producing TNF-α and INF-γ were increased in CD patients, suggesting a new marker of CD-related inflammation [27].

In healthy subjects, ILCs represent about 10% of intraepithelial lymphocytes (IELs) of the gastrointestinal tract, and in the small intestine, they are mainly represented by cytotoxic ILC1 NKp44+ CD127– (ieILC1) and helper ILC1 NKp44– CD127– [5,28]. Uhde et al. demonstrated that in subjects with active or refractory disease, NKp44+ ILC is decreased. At the same time, the frequency of NKp44-ILC is positively correlated with the severity of villous atrophy and epithelial damage. The activation-induced loss of NKp44 by cytotoxic ILC1 determines an increase in IFN-γ levels, thus favoring mucosal damage [28].

Ercolano et al. evaluated the cytokine and ILC profiles in the peripheral blood of children with CD. They found increased circulating levels of IL-12p40, IL-18, and INF-γ associated with normal levels of ILC1 in peripheral blood. Contrary to the intestinal epithelium, where ILC1s are increased, in the peripheral blood, there is an increase in ILC precursors (ILCPs) expressing TG2; these cells bind and internalize gliadin peptides and determine the production of IL-18 and INF-γ, promoting inflammation and the extraintestinal symptoms of CD [29].

NK cells could play a role in the pathogenesis of the disease and their expression could be influenced by diet. However, the results in the literature are often controversial. Indeed, patients with CD have been found to have a lower prevalence of NK, NKT (Natural killer T cell), and invariant NKT (iNKT) cells than healthy subjects do; furthermore, the levels remain altered even after a gluten-free diet [30].

On the contrary, from the studies of Bernardo et al. in 137 CD patients, patients with refractory disease had a significantly reduced number of circulating iNKT cells compared with diet-responsive patients, who had a significantly increased proportion of CD4+ iNKT cells [31]. Further studies on this class of ILC and their correlation with CD and diet may be useful in the future.

## 4. Role of Alarmins in Celiac Disease

Alarmins are endogenous proteins/peptides released following cell damage or in response to immune system induction. They are classified as damage-associated molecular patterns (DAMPs) and interact with chemotactic and pattern recognition receptors (PRRs) to stimulate immune cells into host defense [11].

Alarmins are divided into three groups: (1) granule-derived, (2) nuclear, and (3) cytoplasmic alarmins. Among the granule-derived alarmins, we mainly find α- and β-defensins and cathelicidin (LL37/cathelicidin-related antimicrobial peptide (CRAMP). Also belonging to this category are eosinophil-derived neurotoxin (EDN) and granulysin. Nuclear alarmins include the high-mobility group B1 protein (HMGB-1), the high-mobility group N1 protein (HMGN1), IL-33, and IL-1α, while cytoplasmic alarmins include heat shock proteins (HSP-60, -70, -90 and -96), S100 proteins, ATP, and uric acid [11]. 

According to Perez et al., cell damage and various mechanisms of programmed cell death (PCD) result in the release of alarmins, which may play a role in the expansion of the local inflammatory reaction. Furthermore, alarmins released after PCD stimulate T-cell-induced tissue damage and promote the loss of tolerance to gluten-derived peptides in CD patients [32]. 

To better understand the role of alarmins and the potential link with innate immunity cells, we performed a literature review.

### 4.1. Materials and Methods

We performed research using the international PubMed database and limited the search to articles published between 1 January 2000 and 3 May 2023. Original English-language articles were included; review articles and conference papers were excluded from the search articles outside the scope of the review.

The search was conducted using the keywords: “alarmins” OR “nuclear alarmins” OR “TSLP” OR “IL-33” OR “IL-25” OR “IL-1α” OR “HMGB1” OR “HMGN1” OR “ granule-derived alarmins” OR “defensins” OR “defensins α” OR “defensins β” OR “eosinophil-derived neurotoxin” OR “granulosin” OR “cytoplasmic alarmins” OR “S100 proteins” OR “calprotectin” OR “heat shock proteins ” OR “HSP” OR “ATP” OR “adenosine 5′-triphosphate” OR “uric acid” AND “celiac disease”.

We selected only the articles in which the role of one or more alarmins in the pathogenesis of CD was investigated, grouping studies on humans and animal models. Our search found 19 valuable papers on the potential role of alarmins in the pathogenesis and maintenance of disease. Of these, 18 were studies on humans (serum or biopsy samples). Only one study evaluated β-defensin values in animal models of CD. Six studies concern nuclear alarmins (Thymic stromal lymphopoietin, IL-33, HMBG1), seven on defensins, and six on calprotectin.

### 4.2. Results

#### 4.2.1. Nuclear Alarmins: Thymic Stromal Lymphopoietin (TSLP), IL-33, and HMBG1

TSLP is an IL-7-like cytokine expressed constitutively in the thymus and intestinal epithelial cells. It exerts its biological activities by binding to a heterodimer formed by IL-7 receptor α and TSLP receptor (TSLPR), leading to the activation of the signal transducer and activator of transcription (STAT)5 [33]. At the thymic level, the expression of TSLP favors the differentiation of Treg cells. At the intestinal level, through interaction with the microbiota, it contributes to the maintenance of intestinal homeostasis [34]. TSLP, released by human intestinal epithelial cells (IECs), inhibits IL-12 production by DCs in response to bacteria and drives the development of Th2-polarizing DCs [35,36]. Thus, TSLP has homeostatic activities, interacts with DCs to promote the differentiation of naïve T cells into regulatory T cells (Treg), and blocks the development of Th1 and Th17 [36].

The decrease or increase in the expression of TSLP or its isoforms would determine an imbalance in the intestinal immune system toward the development of a Th1 immune response (in the case of a decrease in the short isoform) or Th2 (when increases the long isoform) [37].

IL-33 is a member of the IL-1 family expressed by endothelial and epithelial cells of barrier tissue [38]. Under healthy conditions, this alarmin maintains barrier function by regulating gene expression as a nuclear protein. At the same time, when released following cell injury, it stimulates the differentiation of T helper cells favoring the activation of the adaptive immune response [39]. IL-33 binds to immune system cells expressing the ST2 receptor (IL-1RL1) [40]. This alarmin potently stimulates ILC2, regulatory T cells (Treg), TH1, CD8+ T, and NK cells [40,41]. Therefore, this alarmin seems essential in tissue and metabolic homeostasis and pathological processes such as infections, inflammations, and tumors [41].

HMGB1 (amphoterin and HMG-1) plays a crucial role in acute and chronic inflammation. HMGB1 is released into the extracellular space by macrophages, monocytes, and endothelial cells after damage to the cell membrane [42].

This alarmin binds to several extracellular receptors, like receptors for advanced glycation end products (RAGE), TLR9, TLR4, TLR2 integrin, T-cell immunoglobulin, and CXCR4 [43].

Under physiological conditions, this alarmin is found within the nucleus of macrophages/monocytes. It is released in response to inflammatory stimuli, promoting the release of TNF-α and the production of pro-inflammatory cytokines and chemokines [44].

In 2016, Biancheri et al. explored mucosal TSLP expression and function in 64 patients with untreated CD and 50 patients with CD after at least 12 months of a gluten-free diet. The authors observed that mucosal TSLP expression, but not TSLPR and IL-7Rα, was reduced in untreated and refractory CD compared to treated CD. Transcripts of both TSLP isoforms were decreased in active CD mucosa. Furthermore, furin has a role in TSLP degradation. Furin expression in untreated Cd mucosa is higher than in control and treated CD [45]. According to Biancheri et al., restoring TSLP might be therapeutically useful, especially in refractory patients with CD.

In 2014, Sziksz et al. investigated the involvement of TSLP and its regulator, the transcription factor peroxisome proliferator-activated receptor gamma (PPARγ), in childhood CD. The authors collected duodenal biopsy specimens from 19 newly diagnosed CD, six children with treated CD, and ten controls. The mucosal expression of TSLP was higher in patients with newly diagnosed CD than in CD patients under a gluten-free diet and controls. PPARγ levels were reduced in patients with CD compared with controls or treated CDs. These results suggest the pathogenetic role of PPARγ and TSLP in CD [46].

More recently, Aksoy et al. evaluated serum TSLP levels in patients with CD. Eighty-nine participants were enrolled: 22 patients with newly diagnosed CD, 20 patients compliant with a gluten-free diet, 32 patients without a gluten-free diet, and 15 healthy controls. Patients with newly diagnosed CD had the highest serum TSLP levels. At the same time, there was no significant difference in serum TSLP levels between patients compliant and noncompliant with gluten-free diet [47].

The interleukin-33/ST2 axis is also involved in the pathogenesis of CD. The possible role of the IL-33/ST2 system in innate immunity of the intestinal mucosa of CD was evaluated by López-Casado et al. in 2017. Both IL-33 and its receptor-soluble ST2 (sST2) serum and tissue levels were significantly higher in patients with CD than in control patients [48]. These findings reflect an active inflammatory state and may represent a potential biomarker for disease activity.

Furthermore, IL-33 and its fragments potentially exacerbate the pro-inflammatory circuit and potentiate the cytotoxic activity of CD8+ T cells in CD, as emerged in a recent study by Perez et al. The authors observed a higher expression of IL-33 in the duodenal mucosa of active CD patients by analyzing duodenal tissues by immunofluorescence and Western blot. Locally digested IL-33 releases active 18/21 kDa fragments, which can contribute to expanding the pro-inflammatory signal. Moreover, increased CD8+ ST2+ T cells and higher transcription factor (T-bet) expression in some ST2+ intraepithelial lymphocytes were observed [49].

In a 2017 multicenter study, Manti et al. demonstrated the potential role of HMGB1 as a biomarker of CD and disease severity. This study observed that the serum levels of HMGB1 were significantly increased in the 49 children with CD compared to the control group (44 children). Furthermore, HMGB1 levels were positively correlated with the disease severity calculated according to the Marsh classification [50].

#### 4.2.2. Granule-Derived Alarmins: Defensins (α, β)

Defensins are a family of cationic antimicrobial peptides consisting of short amino acid chains (~30–40 amino acids) with a β-sheet structure stabilized by three disulfide bonds and divided into three superfamilies: α-defensins, β-defensins, and θ-defensins, the latter not expressed by humans [51,52].

α-Defensins are constitutively expressed by Paneth cells and, in humans, can also be produced by neutrophils. Expression of β-defensins in the intestinal epithelium is inducible, except for human β-defensin 1 (HBD1), which appears to be constitutive [53].

Defensins also play an essential role in maintaining the homeostasis of the intestinal microbiota and participate in innate immunity: HBD1 and HBD2 (human β-defensin 2) have chemo-attractive activity for cells expressing the CC-chemokine 6 (CCR6) receptor, including dendritic cells [53,54].

Mucosal enterocytes, macrophages, and DCs produce β-defensins 2 in the colon [55]. In 2019, Almeida et al. [56] investigated the effects of a gluten diet on fecal quality and quantified blood β-defensin and toll-like receptor 5 (TLR5) gene expression in models of non-human primates, Leontocebus fuscicollis, raised in captivity in the eastern Amazon. The stool consistencies in animals fed a gluten diet were most frequently soft or diarrheic. The expression of β-defensin was increased, returning to normal levels between 30 and 45 days on a gluten-free diet [56].

Given that β-defensins 2 are synthesized by epitheliocytes of the mucosa of the gastrointestinal tract in response to any damaging factor, it is assumed that it can be used as a regional molecular marker of mucous membrane inflammation of the upper intestine [57].

The expressions of different isoforms of defensins throughout the gastrointestinal tract are tissue- and peptide-specific and show high interindividual variability.

In 2000, Frye et al. [58] analyzed mRNA levels of α- and β-defensins and revealed their differential expression in gastrointestinal epithelia. In particular, the HBD1 mRNA was expressed at low levels with little variability throughout the gastrointestinal tract and was detected in all epithelial cells of ileal mucosa. Human α-defensin 5 and 6 (HD5 and HD6, respectively) mRNA expressions were restricted to the intestine, especially in the jejunum and ileum, and displayed high interindividual variability. Biopsies obtained from the duodenum displayed low or no expression of HD5 and HD6 [58].

The expression level changes considerably with the severity of the disease.

The mRNA levels of α-defensins HD5 and HD6 were significantly increased in active CD and returned to normal in treated CD. At the same time, β-defensins HBD-1 and HBD-2 were unaffected in children with untreated, treated, challenged CD, and controls [59].

Furthermore, the behavior of human epithelial HB1 in patients with CD correlated negatively with the degree of villous atrophy (r = −0.64, *p* = 0.019) in a prospective study enrolling 23 adults (13 patients with untreated CD and ten controls) [60].

A subsequent study by Di Sabatino et al. [61] characterized Paneth cells in celiac duodenal mucosa using a multiple histochemical and counting approach and explored Paneth cell function in in vivo and ex vivo experiments by determining mucosal transcripts of HD5 and HD6 in 28 patients with uncomplicated CD, eight patients with complicated CD (e.g., from T-cell lymphoma), and 14 healthy controls. Mucosal HD5 and HD6 were comparable in uncomplicated untreated, treated CD, and control cases [61]. Another study on the gene expression of human β-defensin 1 to 6 (HBD1 to 6) and HD5 and HD6 in mucosal biopsies was performed comparing 11 children with CD with healthy individuals [62]. The study revealed that patients with CD showed different expressions of defensin isoforms: HBD2 was upregulated in the antrum, whereas HBD1 and 4 were downregulated in duodenal biopsies [62]. In the same year, Intrieri et al. [63] analyzed the expression of the HBD1, HBD2, HBD3, and HBD4 genes in patients with CD during the active phase and after remission following a gluten-free diet. The expression of HBD1 was significantly reduced in the duodenum of patients with active CD compared with controls. At the same time, the mRNA levels of the other three defensins did not differ significantly between the two groups. Interestingly, the gluten-free diet only partially restored HBD1 expression compared to celiac-free controls [63]. Also, in 2010, Fernandez-Jimenez et al. [64] evaluated the β-defensin cluster (DEFB4, DEFB103, and DEFB104) by gene-specific, real-time polymerase chain reaction (PCR) in 376 CD patients and 376 controls, revealing that increased copy numbers could protect from CD, possibly by impeding bacterial infiltration more efficiently and preserving gut epithelial integrity. These studies strengthen the concept that a reduced activity of immune peptides may predispose individuals to bacterial proliferation that contributes to the pathogenesis of CD [63,64].

Given that HBD2 is synthesized by epitheliocytes of the mucosa of the gastrointestinal tract in response to any pathogenic noxa, it is possible to speculate that fecal HBD2 could represent a regional biomarker of intestinal inflammation [57,65]. However, Kamilova et al. recently found that fecal HBD2 concentration was higher in the CD group of 76 children than in the healthy control group (99.6 ± 15.5 ng/mL and 64.0 ± 2.4 ng/mL, respectively, *p* < 0.02) [65].

#### 4.2.3. Cytoplasmic Alarmins: Calprotectin

Calprotectin is a cytosolic protein complex consisting of proteins belonging to the S100 leukocyte-calcium-binding protein family. It is constitutively expressed in neutrophils, monocytes, dendritic cells, and activated macrophages; its expression can also be induced by inflammatory processes [66].

S100 proteins, once released, act on different classes of immune cells and other cell types such as endothelial and vascular smooth muscle cells and epithelial cells, thus participating in the inflammatory response [67].

Calprotectin appears to modulate cytoskeletal rearrangements to allow the recruitment of leukocytes, thus promoting chemotaxis and facilitating the transport of arachidonic acid to sites of inflammation [66].

It is still unknown whether fecal calprotectin elevation may be caused by active untreated CD itself or whether it indicates the coexistence of CD and another disease associated with gastrointestinal inflammation. The results of published studies are controversial.

In 2007, Montalto et al. [68] evaluated fecal calprotectin concentrations in 28 untreated celiac patients and correlated them with clinical scores and histological characteristics. For the first time, they showed that fecal calprotectin in untreated celiac patients did not differ significantly from controls [68]. Conversely, another study on 29 newly diagnosed CD children revealed that fecal calprotectin concentration was increased in correspondence to the severity of histopathologic findings and responsive to a gluten-free diet [69].

In 2012, Balamtekın et al. [70] revealed a positive correlation between fecal calprotectin levels and disease activity. The authors concluded that increased fecal calprotectin concentration could be used as a non-invasive marker to aid in diagnosing CD, especially in patients with gastrointestinal presentation. Fecal calprotectin concentration returned to normal on a strict gluten-free diet, suggesting a possible role as a marker of diet adherence and an improvement in gastrointestinal inflammation in children with CD.

Different results were obtained by Capone et al. in 2014 [71]. They concluded that fecal calprotectin could not investigate the subclinical inflammatory changes of active CD in 50 adults with newly diagnosed CD compared to a control group of 50 healthy controls [71]. Similarly, the recent experience by Szaflarska-Popławska et al. [72] suggests that, probably, fecal calprotectin concentration cannot be used as a marker for diagnostics and monitoring of CD independently from the clinical form of the disease and severity of the inflammatory lesions within the small intestine. The authors enrolled 55 children with newly recognized CD and 17 children diagnosed at least one year before on a strict gluten-free diet. Fecal calprotectin was abnormal in 38.2% of patients with newly diagnosed CD and 35.3% of patients on a diet. Moreover, in the group of patients with recently diagnosed CD, a statistically significant relationship was not observed for fecal calprotectin, clinical picture, and severity of small intestinal lesions according to the Marsh classification [72].

## 5. Discussion

The role of ILCs in the pathogenesis of various inflammatory autoimmune and allergic diseases is now well understood in the literature [24].

These cells can mimic the activity of the corresponding T cells, following non-specific stimuli, and can thus determine and maintain inflammation. ILCs have a clear role in intestinal inflammation and IBD [24]; however, their action in CD is still unclear.

There are still few studies in the literature regarding the role of ILCs in CD, and it is necessary to investigate the topic further. However, the results obtained so far agree on the central role of ILC1, whose expression in the lamina propria of the duodenum of patients with active disease is increased compared to other ILC populations. As ILC1 levels are normal in blood serum, the use of these cells as biomarkers of disease or disease severity is useless; instead, it was noted that in peripheral blood, the levels of IL-12p40, IL-18, and INF-γ are increased and correlate positively with the extraintestinal manifestations of the disease.

From the available data, ILCs would have a role in the pathogenesis of CD and the maintenance of inflammation, inducing the release of INF-γ and IL-18. These cytokines, favoring inflammation, determine the damage to the epithelium.

The action of proinflammatory cytokines, such as INF-γ, combined with the low-grade inflammation present at the level of the intestinal mucosa, is responsible for the symptoms and structural alterations found at the level of the intestinal epithelial cells.

Epithelial damage determines the release of alarmins; thus, a link between ILC1 and the alarmins is conceivable and it is responsible for inflammation in patients with CD.

Table 1 summarizes the results of our research, dividing the works into three groups: nuclear alarmins, granule-derived, and cytoplasmic.

The various studies on nuclear alarmins have focused on their role in the pathogenesis and maintenance of the disease and their possible role as biomarkers or therapeutic targets. 

There are still few studies in the literature concerning TSLP and its role in the pathogenesis of CD, and they are sometimes contradictory. Our research has shown that TSLP can increase in the initial stages of the disease and then decrease in the more advanced stages of inflammation. Furthermore, serum TSLP levels appear not to be influenced by the gluten-free diet, and this would indicate the persistence of an underlying inflammation even in patients subjected to the diet, more marked in patients with refractory disease. Intervening on the TSLP pathway could have important therapeutic implications. The efficacy of anti-TSLP antibodies (Tezepelumab) has been extensively demonstrated in treating refractory severe allergic asthma. It may also have a role in treating other pathologies in which Th2 inflammation prevails, such as food allergies and atopic dermatitis [14,73,74]. Biological therapy could also play a role in non-allergic diseases such as cancer, infections, and autoimmune diseases [74,75].

Key roles in inflammation are those of IL-33 and its receptor SST2. The IL-33/SST2 axis appears to be upregulated in patients with active disease and this may exacerbate the pro-inflammatory circuit, enhancing the cytotoxic activity of CD8+ T cells [48,49].

Serum IL-33 levels could be a useful biomarker of disease activity. Furthermore, intervening on the IL-33/SST2 axis could have therapeutic implications.

**Figure 2:**
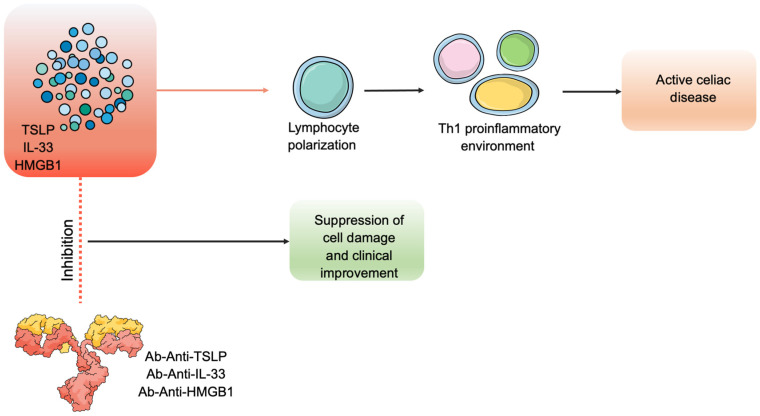
There is evidence about the efficacy of monoclonal antibodies directed against alarmins for treating various pathologies [71,72,73,74,75,76]. They could block the mechanisms underlying the Th1 inflammation that predominates in celiac disease.

HMGB1 is an essential alarmin in inflammatory processes, promoting the release of TNF-α and other pro-inflammatory cytokines. A single study has highlighted its potential role in CD. In fact, in patients with CD, the levels of HMGB1 seem to be positively correlated with damage to the duodenal mucosa [50]. Thus, HMBG1 could be a valuable biomarker of CD severity, reflecting the histopathological pattern of the disease. Some authors have evaluated, on animal models, the possible efficacy of anti-HMGB1 antibodies for treating various neurodegenerative diseases [76,77]. In the future, targeting TSLP, IL-33, or HMGB1 could have therapeutic effects on CD (Figure 2).

Defensins—granule-derived alarmins—are essential in the innate immune response against microorganisms and microbiome maintenance [78]. Defensin deficiency seems to play a pivotal role in the pathogenesis of gastrointestinal diseases, especially Crohn’s disease [79,80]. Furthermore, defensin levels correlate with the activity and severity of several autoimmune-based diseases. For example, HBD2 levels have been found to correlate with the severity of skin manifestations in psoriasis and with disease activity, thus proving to be a promising biomarker of IL-17-driven disease and inflammation [81]. The possibility of using defensins as biomarkers of CD has been evaluated by several authors. A variability in defensin levels depends on the isoform and the disease state [62]. The only study in animal models showed that β-defensin levels were increased in the feces of primates with the disease, normalizing after the diet [56]. Only in one human study were comparable results obtained [65]. Many other authors have found a decreased expression of HBD1 levels in patients with active disease, with an improvement after the gluten-free diet. Furthermore, HBD1 levels negatively correlate with the histological grade of duodenal lesions [60]. The results regarding the value of α-defensins as biomarkers are contradictory. Thus, in CD, HBD1 may be helpful as a biomarker of active disease, disease severity, and response to diet. Some authors have evaluated the possibility of using gene therapy to induce the production of β-defensins for the anticancer effect [82] or therapy with analogs to maintain the balance of systemic homeostasis [83].

There is no utility in measuring fecal calprotectin. The results in the literature could be more comprehensive, and many authors agree on the uselessness of calprotectin as a biomarker of CD both in diagnosis and in response to therapy [68,71,72]. Therefore, there is no link between calprotectin and the pathogenesis of CD. The high calprotectin values found in small populations of patients with CD could be linked to concomitant inflammatory diseases.

## 6. Conclusions

Celiac disease is an autoimmune enteropathy determined by genetic, environmental, and immune factors and driven by Th1 and Th17 inflammation. ILCs, especially ILC1, and alarmins released following damage to the duodenal epithelium appear to be both initiators and maintainers of inflammation.

ILC1s are responsible for intestinal epithelial damage, which causes alarmins’ release. 

There needs to be more studies on ILC1 involved in intestinal inflammation in patients with CD, and it is necessary to focus more attention on the role of these cells.

From the evidence in the literature, nuclear alarmins are clearly involved in the inflammatory process underlying CD. Among these, IL-33 and its receptor SST2 positively correlate with disease severity and could be an excellent biomarker of severity and activity in patients with known disease. TSLP and the IL-33/SST2 axis could also be targeted for future biological therapy (Figure 2). The role of defensins is limited to disease biomarkers, although gene therapy or defensin analogs could have a role in CD therapy. Calprotectin dosage is still useless.

There are promising studies in the literature, but more are needed and they sometimes present conflicting results. Collecting new data regarding the dosage of alarmins in patients with the recently diagnosed disease, in patients with disease treated with a gluten-free diet, and in patients with non-responsive CD to the diet could be helpful in the future to have new non-invasive disease biomarkers and new therapeutic strategies.

Finally, a connection between the ILCs and the alarmins is noticeable.

The study and understanding of this ILC/alarmins axis could be the key to obtaining complementary or replacement therapies for gluten-free diets, which can contribute to improving the quality of life of these patients.

## Figures and Tables

**Table 1:** Studies on alarmins involved in celiac disease.

Alarmins	Author	Age	F/M	N° of Patients	Country	Year [Ref.]
**Studies on humans**						
*Nuclear*						
TSLP	Biancheri et al.	39 years	6/6 *	114	Italy, UK	2016 [45]
	Sziksz et al.	9 years	16/9	25	Hungary	2014 [46]
	Kahramanoğlu Aksoy et al.	41 years	53/20	73	Turkey	2019 [47]
IL-33	López-Casado et al.	6 years	12/8	20	Spain	2017 [48]
	Perez et al.	19 years	39/25	64	Argentina, Chile	2020 [49]
HMGB1	Manti et al.	6 years	16/33	49	Italy	2016 [50]
*Granule-derived*						
Defensin (α, β)	Forsberg et al.	6 years	95/55	150	Sweden	2004 [59]
	Taha et al.	46 years	Unknown	13	UK	2005 [60]
	Di Sabatino et al.	36 years	Unknown	14	Italy	2008 [61]
	Vordenbäumen et al.	4–16 years	8/3	11	Germany	2010 [62]
	Intrieri et al.	35 years	Unknown	21	Italy	2010 [63]
	Fernandez-Jimenez et al.	4 years	241/135	376	Spain	2010 [64]
*Cytoplasmic*						
Calprotectine	Montalto et al.	38 years	12/16	28	Italy	2007 [68]
	Ertekin et al.	6 years	15/14	29	Turkey	2010 [69]
	Balamtekın et al.	8 years	30/34	64	Turkey	2012 [70]
	Capone et al.	Unknown	Unknown	50	Italy	2014 [71]
	Szaflarska-Popławska et al.	10 years	34/38	72	Poland	2020 [72]
**Studies on animal models**						
Β-Defensin	Almeida et al.	\	\	\	Brazil	2019 [56]

* Eight patients with refractory CD and four patients with potential CD.

## Data Availability

Not applicable.
